# Association of Increased Programmed Death Ligand 1 Expression and Regulatory T Cells Infiltration with Higher Hepatocellular Carcinoma Recurrence in Patients with Hepatitis B Virus Pre-S2 Mutant after Curative Surgical Resection

**DOI:** 10.3390/v14061346

**Published:** 2022-06-20

**Authors:** Long-Bin Jeng, Tsai-Chung Li, Shih-Chao Hsu, Chiao-Fang Teng

**Affiliations:** 1 Organ Transplantation Center, China Medical University Hospital, Taichung 404327, Taiwan; longbin.cmuh@gmail.com (L.-B.J.); quietlotus@gmail.com (S.-C.H.); 2 Department of Surgery, China Medical University Hospital, Taichung 404327, Taiwan; 3 School of Medicine, China Medical University, Taichung 404333, Taiwan; 4 Department of Public Health, College of Public Health, China Medical University, Taichung 404333, Taiwan; tcli@mail.cmu.edu.tw; 5 Department of Healthcare Administration, College of Medical and Health Science, Asia University, Taichung 413305, Taiwan; 6 Graduate Institute of Biomedical Sciences, China Medical University, Taichung 404333, Taiwan; 7 Program for Cancer Biology and Drug Development, China Medical University, Taichung 404333, Taiwan; 8 Research Center for Cancer Biology, China Medical University, Taichung 404333, Taiwan

**Keywords:** hepatocellular carcinoma, recurrence, pre-S2 mutant, hepatitis B virus, curative surgical resection, programmed death ligand 1, regulatory T cells

## Abstract

Although surgical resection is available as a potentially curative therapy for hepatocellular carcinoma (HCC), high recurrence of HCC after surgery remains a serious obstacle for long-term patient survival. Therefore, the discovery of valuable prognostic biomarkers for HCC recurrence is urgently needed. Pre-S2 mutant is a mutant form of hepatitis B virus (HBV) large surface protein which is expressed from the HBV surface gene harboring deletion mutations spanning the pre-S2 gene segment. Pre-S2 mutant-positive HCC patients have been regarded as a high-risk population of HCC recurrence after resection surgery and display increased immune checkpoint programmed death ligand 1 (PD-L1) expression and pro-tumor regulatory T cells (Tregs) infiltration in tumor tissues. In this study, the association of higher levels of PD-L1 expression and Tregs infiltration in tumor tissues with post-operative HCC recurrence in pre-S2 mutant-positive HCC patients was evaluated. We found that patients with pre-S2 mutant in combination with higher levels of PD-L1 expression and Tregs infiltration in tumor tissues were independently associated with a higher risk of HCC recurrence (hazard ratio, 4.109; *p* value = 0.0011) and poorer recurrence-free survival (median, 8.2 versus 18.0 months; *p* value = 0.0004) than those of patients with either one or two of these three biomarkers. Furthermore, a combination of pre-S2 mutant, intra-tumoral PD-L1 expression, and tumor-infiltrating Tregs exhibited superior performance in identifying patients at a higher risk of HCC recurrence (area under the receiver operating characteristic curve, 0.8400). Collectively, this study suggests that higher levels of PD-L1 expression and Tregs infiltration in tumor tissues predicted a higher risk of HCC recurrence in pre-S2 mutant-positive HCC patients after curative surgical resection.

## 1. Introduction

As a predominant type of liver cancer, hepatocellular carcinoma (HCC) ranks as the sixth most frequent and third most fatal human cancer worldwide, resulting in over 800,000 deaths per year [1,2,3,4]. Among many therapeutic options for HCC patients, curative surgical resection is considered as one of the most potent treatments [5,6,7,8]. However, the HCC recurrence rate of up to 80% within 5 years after curative surgical resection remains a big challenge, responsible for a 5-year patient survival rate of only 30% [9,10,11]. Therefore, the discovery of potential biomarkers and therapeutic strategies for prediction and prevention of HCC recurrence is urgently needed to improve patient outcomes after resection surgery.

As a major etiological factor of HCC, chronic hepatitis B virus (HBV) infection accounts for nearly 50% of total HCC cases worldwide [4,12,13]. HBV is a lipid-enveloped hepatotropic virus and has a DNA genome incorporated into the viral nucleocapsid [14,15]. The HBV surface gene consists of three gene segments, namely the pre-S1, the pre-S2, and the S, and encodes three different sizes of surface proteins (small, middle, and large) from the S segment, the pre-S2 and S segments, and all three segments, respectively, which collectively constitute the envelope proteins of viral particles. Moreover, integration of HBV DNA into the host cell genomes is a frequent event occurred in HBV-related HCC, resulting in the induction of insertional mutagenesis and genomic instability and the persistent expression of viral proteins, particularly HBV surface and X proteins [16,17,18].

Several naturally occurring in-frame deletion mutations spanning the pre-S2 gene segment (predominantly nucleotides 1 to 54) with or without point mutations at the start codon of the pre-S2 gene segment of HBV surface gene have been clinically identified, leading to the expression of a mutant form of HBV large surface protein called the pre-S2 mutant and defective synthesis of the middle surface protein [19,20,21]. Because the region of nucleotides 1 to 54 in the pre-S2 gene segment overlaps the nucleocapsid-binding domain of HBV large surface protein, deletion of this region abrogates the assembly and secretion of viral particles [22,23]. Moreover, the region of nucleotides 1 to 54 in the pre-S2 gene segment coincides with the neutralization B cell and cytotoxic T cell epitopes of HBV large surface protein [24,25], suggesting that pre-S2 mutant may represent an immune escape mutant in chronic HBV infection. It has been well demonstrated that pre-S2 mutant is an important HBV oncoprotein that accumulates in the endoplasmic reticulum (ER) to induce ER stress and through which multiple oncogenic signaling pathways involved in cell proliferation are activated, including cell cycle progression, survival, metabolism, and genomic instability, to promote hepatocyte malignant transformation and HCC development [19,26,27,28,29,30,31,32,33,34,35,36,37,38]. Targeting at the pre-S2 mutant-activated oncogenic signals exhibits a preventive effect on HCC formation [31,39,40,41]. Furthermore, in patients with chronic hepatitis B and HBV-related HCC, the presence of pre-S2 mutant in blood and liver tissues serves as an independent biomarker for higher incidences of HCC development and recurrence after curative surgical resection, respectively [42,43,44,45,46,47,48,49]. Therefore, there is an urgent need to develop potential therapeutic strategies for treating the pre-S2 mutant-positive high-risk patient populations.

As a key hallmark of cancer, tumor cells can evade host immune surveillance through many mechanisms such as induction of immune checkpoint programmed death ligand 1 (PD-L1) expression and activation of pro-tumor regulatory T cells (Tregs) function to suppress cytotoxic T cells (CTLs)-mediated anti-tumor immune responses [50,51,52,53,54,55]. Multiple lines of evidence indicate that a higher level of PD-L1 expression or Tregs infiltration in tumor tissues of HCC patients is associated with poorer prognosis after curative surgical resection [56,57,58,59,60,61,62,63,64,65]. In addition, the pre-S2 mutant-positive HCC patients have been shown to display increased expression of intra-tumoral PD-L1 and elevated number of tumor-infiltrating Tregs compared with pre-S2 mutant-negative HCC patients [66,67]. However, whether the higher levels of PD-L1 expression and Tregs infiltration in tumor tissues of pre-S2 mutant-positive HCC patients predict a higher risk of HCC recurrence after curative surgical resection remain to be clarified.

In this study, 40 HBV-related HCC patients were enrolled and classified into pre-S2 mutant-positive and -negative groups of patients. The expression of PD-L1 and infiltration of Tregs in tumor tissues of patients were determined. The association between the levels of PD-L1 expression and Tregs infiltration in tumor tissues and the rate of HCC recurrence after curative surgical resection in pre-S2 mutant-positive HCC patients were statistically analyzed.

## 2. Materials and Methods

### 2.1. Collection of Patient Specimens

Pre-operative plasma and formalin-fixed and paraffin-embedded (FFPE) liver tissues were retrospectively collected from 40 HBV-related HCC patients who were positive for serum HBV surface antigen (HBsAg) and underwent curative surgical resection at China Medical University Hospital (Taichung, Taiwan) under the approval of the China Medical University & Hospital Research Ethics Committee (protocol No. CMUH107-REC1-080) from January 2006 to July 2017. Clinicopathological data of the patients were also retrieved from the hospital records. All research was conducted in accordance with the guidelines of the 1975 Declaration of Helsinki and informed consent was obtained from all participants.

### 2.2. Detection of Deletion Mutations Spanning the Pre-S2 Gene Segment in Plasma

Deletion mutations spanning the pre-S2 gene segment of HBV surface gene were detected in plasma of HBV-related HCC patients by using a next-generation sequencing (NGS)-based approach as previously described [68,69,70]. Briefly, plasma DNA was extracted by using the DNeasy Blood Kit (Qiagen, Valencia, CA, USA) and served as a template for amplification of the entire HBV pre-S gene (comprising the pre-S1 and pre-S2 gene segments) by polymerase chain reaction (PCR) using the high-fidelity Platinum SuperFi DNA polymerase (Invitrogen, Carlsbad, CA, USA). The resulting pre-S gene PCR products were subsequently analyzed by NGS using the NextSeq 500 System supplemented with the bcl2fastq Conversion Software v2.20 (Illumina, San Diego, CA, USA) following the manufacturer’s instructions. Pre-S2 mutant-positive and -negative patients were defined according to the presence and absence of deletion mutations spanning the pre-S2 gene segment in plasma, respectively (Table S1).

### 2.3. Detection of Intratumoral PD-L1 and Tumor-Infiltrating Tregs in Liver Tissues

Intra-tumoral PD-L1 expression and tumor-infiltrating Tregs were detected in FFPE liver tissues of HBV-related HCC patients by using a fluorescent immunohistochemistry (IHC) staining-based approach as previously described [66,67]. Briefly, 4-μm-thick tissue sections were incubated with the primary antibodies mouse anti-PD-L1 (AM26531AF-N; Acris, Hiddenhausen, Germany) for detection of PD-L1 or rabbit anti-CD4 (ab133616; Abcam, Cambridge, UK) together with mouse anti-CD25 (Leica Biosystems, Buffalo Grove, IL, USA) for detection of Tregs, followed by the secondary antibodies Alexa Fluor 555-conjugated anti-mouse IgG (A-21424; Invitrogen) and/or Alexa Fluor 488-conjugated anti-rabbit IgG (A11008; Invitrogen). Nuclei were counterstained with 4ʹ, 6-diamidino-2-phenylindole (DAPI; Invitrogen). Additionally, histopathology of the tissue sections was examined by hematoxylin and eosin (H&E) staining to define the tumor regions. Whole-slide images of the fluorescent IHC- and H&E-stained tissue sections were obtained by using the 3DHistech Pannoramic SCAN II scanner (3DHistech, Budapest, Hungary) and Aperio CS2 scanner (Leica Biosystems), respectively. Total number of PD-L1-expressing cells or Tregs (CD4 and CD25 double-expressing cells) in 10 microscopic fields (original magnification, ×40; area, 1.566 mm^2^ per field) independently selected with the most abundant PD-L1-expressing cells or Tregs in the tumor region of each patient’s tissue section was counted and presented as the density (number of cells per mm^2^; cells/mm^2^) for statistical analysis (Figure S1).

### 2.4. Statistical Analysis

Clinicopathological correlation was analyzed by the chi-square test. Univariate and multivariate recurrence-free survival (RFS) analyses were conducted by the Cox proportional-hazards regression model. RFS curves were generated by the Kaplan–Meier method and compared by the log-rank test. Receiver operating characteristic (ROC) curves were established to differentiate patients with HCC recurrence from patients without HCC recurrence and the area under the ROC curve (AUC) was compared by the Hanley–McNeil test. A *p* value of < 0.05 was considered statistically significant.

## 3. Results

### 3.1. Association of HBV Pre-S2 Mutant-Positive HCC Patients with Higher Levels of PD-L1 Expression and Tregs Infiltration in Tumor Tissues

The clinicopathological profiles and correlation of the 40 HBV-related HCC patients enrolled in this study are summarized in Table 1. Among all patients, there were 34 men and six women and the median age was 40 years (range, 28–78). Of these patients, 36 out of 40 had detectable HBV DNA at a median level of 5.0 log_10_ copies/mL (range, 1.5–8.2) and 34 of 40 were negative for HBV e antigen (HBeAg). All patients had a median tumor size of 4.0 cm (range, 1.5–35.0) and received curative surgical resection, among whom 25 patients suffered post-operative HCC recurrence with a median RFS of 11.2 months (range, 1.5–72.3). According to fluorescent IHC staining-based analysis, the median densities of PD-L1-expressing cells and Tregs in tumor tissues of all patients were 14.37 cells/mm^2^ (range, 3.45–32.38) and 8.27 cells/mm^2^ (range, 0.89–19.60), respectively (Table 2). In addition, by NGS-based analysis, all patients were divided into 19 pre-S2 mutant-negative and 21 pre-S2 mutant-positive patients. The correlation between pre-S2 mutant and PD-L1 expression or Tregs infiltration in tumor tissues or other clinicopathological characteristics was evaluated and showed that the pre-S2 mutant-positive HCC patients were significantly associated with higher densities of PD-L1-expressing cells (median (range), 18.20 (6.96–32.38) versus 7.09 (3.45–18.07) cells/mm^2^; *p* value < 0.0001) and Tregs (median (range), 12.84 (8.05–19.60) versus 2.17 (0.89–6.96) cells/mm^2^; *p* value < 0.0001) in tumor tissues than the pre-S2 mutant-negative HCC patients (Table 2). The densities of PD-L1-expressing cells and Tregs in tumor tissues were further divided into high and low densities by using the median density as a cut-off value. Up to 18 (85%) and 20 (95%) of 21 pre-S2 mutant-positive patients had high densities of PD-L1-expressing cells and Tregs in tumor tissues but only two (10%) and none of 19 pre-S2 mutant-negative patients had high densities of PD-L1-expressing cells and Tregs, respectively (Table 2).

### 3.2. Association of Higher Levels of PD-L1 Expression and Tregs Infiltration in Tumor Tissues of HBV Pre-S2 Mutant-Positive HCC Patients with a Higher Risk of HCC Recurrence and Poorer RFS after Curative Surgical Resection

**Figure 1:**
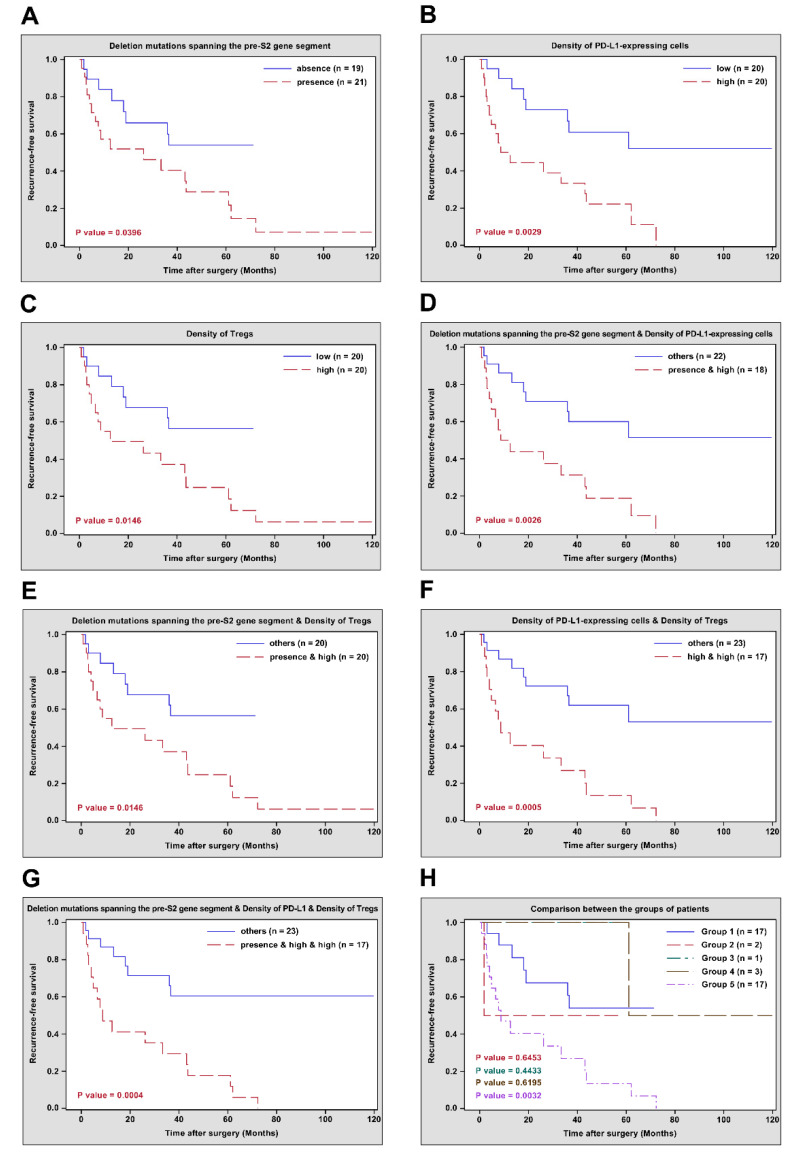
RFS curves in different groups of HBV-related HCC patients after curative surgical resection. (**A**) RFS curves in patients with presence versus absence of deletion mutations spanning the pre-S2 gene segment. (**B**) RFS curves in patients with high versus low density of PD-L1-expressing cells. (**C**) RFS curves in patients with high versus low density of Tregs. (**D**) RFS curves in patients with the presence of deletion mutations spanning the pre-S2 gene segment and high density of PD-L1-expressing cells versus other combinations. (**E**) RFS curves in patients with the presence of deletion mutations spanning the pre-S2 gene segment and high density of Tregs versus other combinations. (**F**) RFS curves in patients with high densities of PD-L1-expressing cells and Tregs versus other combinations. (**G**) RFS curves in patients with the presence of deletion mutations spanning the pre-S2 gene segment and high densities of PD-L1-expressing cells and Tregs versus other combinations. (**H**) RFS curves in the Groups 2, 3, 4, and 5 of patients versus the Group 1 of patients, respectively. The number of patients in each group and *p* values between different groups of patients were indicated in the plots. Abbreviations: RFS, recurrence-free survival; HBV, hepatitis B virus; HCC, hepatocellular carcinoma; PD-L1, programmed death ligand 1; Tregs, regulatory T cells; n, number.

The correlation of pre-S2 mutant, intra-tumoral PD-L1 expression, tumor-infiltrating Tregs, and other clinicopathological characteristics with HCC recurrence after curative surgical resection was assessed by univariate and multivariate analyses as well as RFS curves. In univariate analysis, the presence of deletion mutations spanning the pre-S2 gene segment in plasma or a high density of PD-L1-expressing cells or Tregs in tumor tissues was significantly associated with a higher risk of HCC recurrence (hazard ratio (HR) (95% confidence interval (CI)), 2.378 (1.015–5.573), *p* value = 0.0461; 3.379 (1.446–7.900), *p* value = 0.0049; 2.769 (1.180–6.498), *p* value = 0.0193, respectively) and poorer RFS (median (range), 7.7 (0.8–72.3) versus 18.5 (1.8–43.7) months, *p* value = 0.0396; 7.1 (0.8–72.3) versus 19.0 (2.1–61.1) months, *p* value = 0.0029; 8.2 (0.8–72.3) versus 18.0 (1.8–43.7) months, *p* value = 0.0146, respectively) than those of the absence of deletion mutations spanning the pre-S2 gene segment or a low density of PD-L1-expressing cells or Tregs (Table 3 and Figure 1A–C). A combination of the presence of deletion mutations spanning the pre-S2 gene segment and a high density of PD-L1-expressing cells or Tregs or a combination of high densities of PD-L1-expressing cells and Tregs was significantly associated with a higher risk of HCC recurrence (HR (95% CI), 3.315 (1.452–7.570), *p* value = 0.0044; 2.769 (1.180–6.498), *p* value = 0.0193; 3.928 (1.716–8.995), *p* value = 0.0012, respectively) and poorer RFS (median (range), 7.7 (0.8–72.3) versus 18.5 (1.8–61.1) months, *p* value = 0.0026; 8.2 (0.8–72.3) versus 18.0 (1.8–43.7) months, *p* value = 0.0146; 8.2 (0.8–72.3) versus 18.0 (1.8–61.1) months, *p* value = 0.0005, respectively) than those of other combinations (Table 3 and Figure 1D–F). Moreover, a combination of the presence of deletion mutations spanning the pre-S2 gene segment and high densities of PD-L1-expressing cells and Tregs was significantly associated with a higher risk of HCC recurrence (HR (95% CI), 4.109 (1.763–9.572), *p* value = 0.0011) and poorer RFS (median (range), 8.2 (0.8–72.3) versus 18.0 (1.8–43.7) months, *p* value = 0.0004) than those of other combinations (Table 3 and Figure 1G). When all patients were further classified into distinct groups according to their status at deletion mutations spanning the pre-S2 gene segment and densities of PD-L1-expressing cells and Tregs, 17 patients fell into Group 1 (absence of deletion mutations spanning the pre-S2 gene segment and low densities of PD-L1-expressing cells and Tregs), two patients fell into Group 2 (absence of deletion mutations spanning the pre-S2 gene segment, high density of PD-L1-expressing cells, and low density of Tregs), one patient fells into Group 3 (presence of deletion mutations spanning the pre-S2 gene segment, high density of PD-L1-expressing cells, and low density of Tregs), three patients fell into Group 4 (presence of deletion mutations spanning the pre-S2 gene segment, low density of PD-L1-expressing cells, and high density of Tregs), and 17 patients fell into Group 5 (presence of deletion mutations spanning the pre-S2 gene segment and high densities of PD-L1-expressing cells and Tregs). Only Group 5, rather than other groups of patients, had a significantly higher risk of HCC recurrence (HR (95% CI), 3.593 (1.456–8.862), *p* value = 0.0055) and poorer RFS (median (range), 8.2 (0.8–72.3) versus 19.0 (3.0–43.7) months, *p* value = 0.0032) than Group 1 (Table 3 and Figure 1H). Furthermore, in multivariate analysis, a high density of PD-L1-expressing cells, a combination of high densities of PD-L1-expressing cells and Tregs, and a combination of the presence of deletion mutations spanning the pre-S2 gene segment and high densities of PD-L1-expressing cells and Tregs, were shown as independent biomarkers for predicting a higher risk of HCC recurrence (HR (95% CI), 2.663 (1.069–6.632), *p* value = 0.0354; 2.580 (1.022–6.516), *p* value = 0.0449; 3.163 (1.309–7.644), *p* value = 0.0105, respectively) (Table 3). The Group 5 of patients were independently associated with a higher risk of HCC recurrence (HR (95% CI), 2.837 (1.048–7.679), *p* value = 0.0401) than that of the Group 1 of patients (Table 3).

### 3.3. Superior Performance of HBV Pre-S2 Mutant in Combination with Higher Levels of PD-L1 Expression and Tregs Infiltration in Tumor Tissues in Identifying Patients at Higher Risk of HCC Recurrence after Curative Surgical Resection

**Figure 2:**
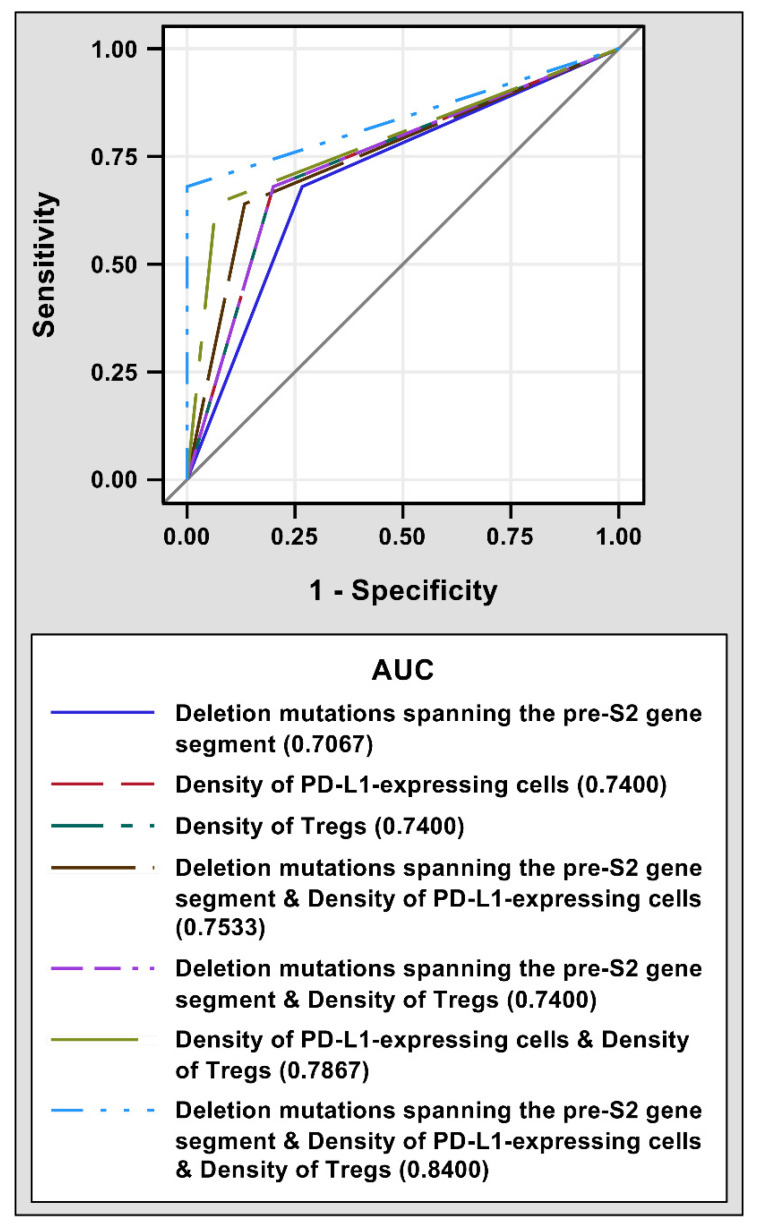
ROC curves of different prognostic biomarkers for HCC recurrence after curative surgical resection. 25 patients with and 15 patients without post-operative HCC recurrence were analyzed. The AUCs for deletion mutations spanning the pre-S2 gene segment (solid dark blue line), density of PD-L1-expressing cells (dashed red line), density of Tregs (dashed dark green line), a combination of deletion mutations spanning the pre-S2 gene segment and density of PD-L1-expressing cells (dashed brown line), a combination of deletion mutations spanning the pre-S2 gene segment and density of Tregs (dashed purple line), a combination of density of PD-L1-expressing cells and density of Tregs (dashed light green line), and a combination of deletion mutations spanning the pre-S2 gene segment, density of PD-L1-expressing cells, and density of Tregs (dashed light blue line) were shown in the bottom of the plot. Abbreviations: ROC, receiver operating characteristic; HCC, hepatocellular carcinoma; AUC, area under the ROC curve; PD-L1, programmed death ligand 1; Tregs, regulatory T cells.

The prognostic performance of pre-S2 mutant, intra-tumoral PD-L1 expression, tumor-infiltrating Tregs, and a combination of either two or all of these three biomarkers for HCC recurrence after curative surgical resection was evaluated by ROC curves. As shown in Figure 2, a combination of deletion mutations spanning the pre-S2 gene segment, density of PD-L1-expressing cells, and density of Tregs had the highest AUC (0.8400, 95% CI 0.7467–0.9333) followed by a combination of density of PD-L1-expressing cells and density of Tregs (0.7867, 95% CI 0.6705–0.9028), a combination of deletion mutations spanning the pre-S2 gene segment and density of PD-L1-expressing cells (0.7533, 95% CI 0.6224–0.8843), a combination of deletion mutations spanning the pre-S2 gene segment and density of Tregs (0.7400, 95% CI 0.5997–0.8803), density of PD-L1-expressing cells (0.7400, 95% CI 0.5997–0.8803), density of Tregs (0.7400, 95% CI 0.5997–0.8803), and deletion mutations spanning the pre-S2 gene segment (0.7067, 95% CI 0.5579–0.8554).

## 4. Discussion

Although surgical resection is considered as a potentially curative treatment for HCC, high recurrence rate of HCC after surgery is still a significant threat, responsible for poor patient outcomes [9,10,11]. Therefore, the development of useful biomarkers for identifying patients at a higher risk of post-operative HCC recurrence for better surveillance and management remains a key goal to improve patient survival. In this study, we found that higher levels of PD-L1 expression and Tregs infiltration in tumor tissues of HBV pre-S2 mutant-positive HCC patients were associated with a higher risk of HCC recurrence and poorer RFS after curative surgical resection. Furthermore, a combination of the presence of deletion mutations spanning the pre-S2 gene segment and high densities of PD-L1-expressing cells and Tregs was validated as an independent prognostic biomarker with better performance in predicting a higher risk of post-operative HCC recurrence than that of either one or a combination of either two of these three biomarkers.

As an important HBV oncoprotein, pre-S2 mutant can induce HCC development through activation of multiple oncogenic signaling pathways to promote hepatocyte malignant transformation, including endoplasmic reticulum stress, genomic instability, anchorage-independent cell growth, cell proliferation, cell cycle progression, cell survival, and glucose and lipid metabolism. [19,26,27,28,29,30,31,32,33,34,35,36,37,38]. Patients with pre-S2 mutant have been regarded as a high-risk population for HCC recurrence after curative surgical resection and the presence of deletion mutations spanning the pre-S2 gene segment in plasma represents an independent biomarker for predicting a higher risk of HCC recurrence after surgery [46,47,48,49]. However, there is still room for improvement in the prognostic performance of pre-S2 mutant serving as a biomarker for HCC recurrence. Recently, it has been shown that the presence of deletion mutations spanning the pre-S2 gene segment together with low serum albumin level exhibits better prognostic performance for HCC recurrence after curative surgical resection than that of either biomarker alone [71]. In this study, we further identified that patients with pre-S2 mutant in combination with higher levels of PD-L1 expression and Tregs infiltration in tumor tissues were associated with a higher risk of post-operative HCC recurrence than that of patients with either one or two of these three biomarkers.

PD-L1 and Tregs play important roles in the evasion of tumor cells from host immune surveillance in the tumor microenvironment, contributing to cancer development [50,51]. As one of the immune checkpoint molecules, PD-L1 expressed on tumor cells interacts with its receptor programmed death 1 (PD-1) expressed on CTLs, leading to inhibition of CTLs-mediated anti-tumor immune responses [52,53]. As one of the tumor-infiltrating lymphocytes, Tregs exert immunosuppressive and pro-tumor functions through suppression of the anti-tumor activities of CTLs, such as inhibition of their activation, proliferation, and secretion of cytotoxic proteins and cytokines [54,55]. Moreover, PD-L1 plays an essential role in promoting the development, expansion, and suppressive function of Tregs [72,73,74]. Consistent with the importance of PD-L1 and Tregs in tumor progression, a higher level of PD-L1 expression or Tregs infiltration in tumor tissues has been associated with poorer prognosis in HCC patients after curative surgical resection [56,57,58,59,60,61,62,63,64,65]. In this study, we further showed that a combination of higher levels of PD-L1 expression and Tregs infiltration in tumor tissues was associated with a higher risk of HCC recurrence and poorer RFS than those of either biomarker alone in pre-S2 mutant-positive HCC patients after curative surgical resection. Considering that the therapeutics targeting PD-L1 and Tregs either alone or in combination show great promise in the treatment of many cancers including HCC [75,76,77,78,79,80], the results of this study may therefore not only suggest the levels of PD-L1 expression and Tregs infiltration in tumor tissues as valuable prognostic biomarkers but also support PD-L1- and Tregs-targeted therapies as potential therapeutic options for pre-S2 mutant-positive HCC patients after curative surgical resection although the efficacy remains to be validated.

There are some limitations in this study. First, the number of patients analyzed is too small. Although the clinicopathological profiles of the patients correspond with the representative characteristics of a large population of HCC patients in Taiwan [81], a greater number of patients from different clinical centers is required to further evaluate the results of this study and its application in clinical practice. Next, the HBV genotypes of the patients enrolled in this study are mainly B and C. Although it has been shown that genotypes B and C are the most prevalent HBV strains in Asian countries including Taiwan [82] and there is no significant difference in the deletion patterns of pre-S2 gene segment between these two genotypes [83], whether the results of this study are applicable to other genotypes remains to be clarified. In addition, the underlying mechanisms of the close association between pre-S2 mutant and high densities of PD-L1-expressing cells and Tregs in HCC tissues remain unclear. Some overlap has been observed between the pre-S2 mutant-activated signaling pathways and the signaling pathways upregulating PD-L1 and Tregs such as vascular endothelial growth factor-A, transforming growth factor-β, mammalian target of rapamycin, and Myc [30,31,84,85,86,87], supporting that pre-S2 mutant may play a potential role in induction of intratumoral PD-L1 expression and Tregs infiltration.

## 5. Conclusions

In this study, we demonstrated that a combination of HBV pre-S2 mutant and higher levels of PD-L1 expression and Tregs infiltration in tumor tissues was an independent prognostic biomarker with greater performance in identifying patients at a higher risk of HCC recurrence after curative surgical resection than that of either one or a combination of either two of these three biomarkers.

## Figures and Tables

**Table 1:** Clinicopathological profiles and correlation of the 40 HBV-related HCC patients.

Clinicopathological Characteristics ^a^	Total Patients (No. of Patients (Median, Range))	Pre-S2 Mutant-Negative Patients (No. of Patients (Median, Range))	Pre-S2 Mutant-Positive Patients (No. of Patients (Median, Range))	*p* Value ^b^
Age (years)	40 (54.0, 28–78)	19 (57.0, 38–75)	21 (52.0, 28–78)	
>50	29 (58.0, 51–78)	16 (57.5, 51–75)	13 (58.0, 52–78)	0.0853
≤50	11 (47.0, 28–49)	3 (39.0, 38–49)	8 (47.5, 28–49)	
Gender	40	19	21	
men	34	16	18	0.3358
women	6	3	3	
Smoking	40	19	21	
yes	14	7	7	0.2525
no	26	12	14	
Alcohol	40	19	21	
yes	9	2	7	0.0727
no	31	17	14	
HBeAg	40	19	21	
positive	6	2	4	0.2666
negative	34	17	17	
HBV genotype	40	19	21	
B	31	15	16	0.2884
C	9	4	5	
HBV DNA (log_10_ copies/mL)	36 (5.0, 1.5–8.2)	18 (5.1, 2.0–8.2)	18 (4.7, 1.5–7.7)	
>4	24 (5.7, 4.1–8.2)	12 (6.0, 4.4–8.2)	12 (5.4, 4.1–7.7)	0.2753
≤4	12 (2.6, 1.5–3.8)	6 (2.6, 2.0–3.7)	6 (2.9, 1.5–3.8)	
Albumin (g/dL)	40 (3.8, 2.0–4.9)	19 (3.9, 2.0–4.5)	21 (3.7, 2.4–4.9)	0.0775
>3.8	18 (4.2, 3.9–4.9)	11 (4.2, 3.9–4.5)	7 (4.0, 3.9–4.9)	
≤3.8	22 (3.4, 2.0–3.8)	8 (3.1, 2.0–3.8)	14 (3.4, 2.4–3.7)	
AST (U/L)	40 (52.5, 14–290)	19 (50.0, 20–238)	21 (59.0, 14–290)	
>34	32 (64.5, 35–290)	17 (52.0, 35–238)	15 (98.0, 42–290)	0.1207
≤34	8 (25.0, 14–34)	2 (22.0, 20–24)	6 (27.5, 14–34)	
ALT (U/L)	40 (55.5, 13–292)	19 (57.0, 30–139)	21 (50.0, 13–292)	
>40	26 (80.0, 42–292)	13 (96.0, 42–139)	13 (65.0, 43–292)	0.2379
≤40	14 (33.0, 13–40)	6 (35.5, 30–40)	8 (29.0, 13–38)	
AFP (ng/mL)	40 (37.7, 1.4–4550.0)	19 (26.7, 1.4–3266.0)	21 (108.4, 2.7–4550.0)	
>400	15 (823.6, 412.3–4550.0)	5 (670.5, 412.3–3266.0)	10 (838.4, 461.7–4550.0)	0.102
≤400	25 (20.2, 1.4–280.7)	14 (10.7, 1.4–280.7)	11 (23.6, 2.7–207.0)	
Tumor size (cm)	40 (4.0, 1.5–35.0)	19 (4.5, 1.5–35.0)	21 (3.5, 1.5–15.0)	
>5	14 (10.0, 5.5–35.0)	7 (8.1, 5.5–35.0)	7 (10.0, 6.5–15.0)	0.2525
≤5	26 (3.0, 1.5–11.0)	12 (2.7, 1.5–5.0)	14 (3.0, 1.5–11.0)	
Tumor encapsulation	39	19	20	
yes	30	16	14	0.1772
no	9	3	6	
Satellite nodule	40	19	21	
yes	7	2	5	0.1866
no	33	17	16	
Lymph node involvement	40	19	21	
yes	6	4	2	0.2121
no	34	15	19	
Portal vein thrombosis	40	19	21	
yes	1	1	0	0.475
no	39	18	21	
Vascular invasion	40	19	21	
yes	18	8	10	0.2351
no	22	11	11	
Distant metastasis	40	19	21	
yes	4	1	3	0.2765
no	36	18	18	
Steatosis grade	33	19	14	
2/3	1	1	0	0.5758
0/1	32	18	14	
Metavir inflammation score	35	18	17	
2/3	1	0	1	0.4857
0/1	34	18	16	
Ishak fibrosis score	39	19	20	
4/5/6	15	7	8	0.2525
0/1/2/3	24	12	12	
Child-Pugh cirrhosis score	40	19	21	
B/C	10	4	6	0.2481
A	30	15	15	
CLIP score	40	19	21	
4/5/6	2	2	0	0.2192
0/1/2/3	38	17	21	
BCLC stage	40	19	21	
C/D	8	3	5	0.2666
A/B	32	16	16	
AJCC TNM stage	40	19	21	
IIIA/IIIB/IIIC/IVA/IVB	9	4	5	0.2884
I/II	31	15	16	

^a^ Only patients with available data were analyzed. ^b^ *p* value was determined between the pre-S2 mutant-positive and -negative patients by the chi-square test. Abbreviations: HBV, hepatitis B virus; HCC, hepatocellular carcinoma; PD-L1, programmed death ligand 1; Tregs, regulatory T cells; HBeAg, hepatitis B e antigen; AST, aspartate aminotransferase; ALT, alanine aminotransferase; AFP, alpha-fetoprotein; CLIP, Cancer of the Liver Italian Program; BCLC, Barcelona Clinic Liver Cancer; AJCC, American Joint Committee on Cancer; TNM, tumor-node-metastasis.

**Table 2:** Densities of PD-L1-expressing cells and Tregs in tumor tissues of the 40 HBV-related HCC patients.

Clinicopathological Characteristics ^a^	Total Patients (No. of Patients (Median, Range))	Pre-S2 Mutant-Negative Patients (No. of Patients (Median, Range))	Pre-S2 Mutant-Positive Patients (No. of Patients (Median, Range))	*p* Value ^b^
Density of PD-L1-expressing cells ^c^	40 (14.37, 3.45–32.38)	19 (7.09, 3.45–18.07)	21 (18.20, 6.96–32.38)	
high	20 (19.80, 14.81–32.38)	2 (16.83, 15.58–18.07)	18 (21.49, 14.81–32.38)	<0.0001 ***
low	20 (7.02, 3.45–13.92)	17 (6.83, 3.45–12.39)	3 (10.34, 6.96–13.92)	
Density of Tregs ^d^	40 (8.27, 0.89–19.60)	19 (2.17, 0.89–6.96)	21 (12.84, 8.05–19.60)	
high	20 (12.90, 8.49–19.60)	0	20 (12.90, 8.49–19.60)	<0.0001 ***
low	20 (2.20, 0.89–8.05)	19 (2.17, 0.89–6.96)	1 (8.05, 8.05–8.05)	

^a^ Only patients with available data were analyzed. ^b^ *p* value was determined between the pre-S2 mutant-positive and -negative patients by the chi-square test. ^c^ The density of PD-L1-expressing cells in tumor tissues above the median density of 14.37 cells/mm^2^ was defined as high density. ^d^ The density of Tregs in tumor tissues above the median density of 8.27 cells/mm^2^ was defined as high density. ***, *p* value < 0.001. Abbreviations: PD-L1, programmed death ligand 1; Tregs, regulatory T cells; HBV, hepatitis B virus; HCC, hepatocellular carcinoma.

**Table 3:** Univariate and multivariate recurrence-free survival analyses of the 40 HBV-related HCC patients.

Clinicopathological Characteristics ^a^ (Comparison, No. of Patients)	Univariate Analysis			Multivariate Analysis		
	HR	95% CI	*p* Value ^b^	HR	95% CI	*p* Value ^b^
Deletion mutations spanning the pre-S2 gene segment (presence, 21 vs. absence, 19)	2.378	1.015–5.573	0.0461 *	2.095	0.833–5.271	0.1163 ^c^
Density of PD-L1-expressing cells (high, 20 vs. low, 20)	3.379	1.446–7.900	0.0049 **	2.663	1.069–6.632	0.0354 *^,d^
Density of Tregs (high, 20 vs. low, 20)	2.769	1.180–6.498	0.0193 *	2.270	0.884–5.827	0.0884 ^e^
Deletion mutations spanning the pre-S2 gene segment & Density of PD-L1-expressing cells (presence & high, 18 vs. others, 22)	3.315	1.452–7.570	0.0044 **	2.318	0.953–5.636	0.0636 ^f^
Deletion mutations spanning the pre-S2 gene segment & Density of Tregs (presence & high, 20 vs. others, 20)	2.769	1.180–6.498	0.0193 *	2.270	0.884–5.827	0.0884 ^g^
Density of PD-L1-expressing cells & Density of Tregs (high & high, 17 vs. others, 23)	3.928	1.716–8.995	0.0012 **	2.580	1.022–6.516	0.0449 *^,h^
Deletion mutations spanning the pre-S2 gene segment & Density of PD-L1-expressing cells & Density of Tregs (presence & high, 17 & high vs. others, 23)	4.109	1.763–9.572	0.0011 **	3.163	1.309–7.644	0.0105 *^,i^
Deletion mutations spanning the pre-S2 gene segment & Density of PD-L1-expressing cells & Density of Tregs Group 2 (absence & high & low, 2) vs. Group 1 (absence & low & low, 17) Group 3 (presence & high & low, 1) vs. Group 1 (absence & low & low, 17) Group 4 (presence & low & high, 3) vs. Group 1 (absence & low & low, 17) Group 5 (presence & high & high, 17) vs. Group 1 (absence & low & low, 17)						
					
					
	1.866	0.228–15.311	0.5611	4.746	0.490–46.006	0.1791 ^j^
					
	0.716	0.212–2.970	0.9919	0.899	0.303–7.431	0.9928 ^j^
					
	0.561	0.068–4.650	0.5922	0.996	0.112–8.885	0.9970 ^j^
					
	3.593	1.456–8.862	0.0055 **	2.837	1.048–7.679	0.0401 *^,j^
Age (years) (>50, 29 vs. ≤50, 11)	0.556	0.243–1.271	0.1640			
Gender (men, 34 vs. women, 6)	0.500	0.185–1.352	0.1723			
Smoking (yes, 14 vs. no, 26)	0.374	0.139–1.005	0.0512			
Alcohol (yes, 9 vs. no, 31)	1.256	0.495–3.192	0.6313			
HBeAg (positive, 6 vs. negative, 34)	2.670	0.978–7.288	0.0552			
HBV genotype (B, 31 vs. C, 9)	0.683	0.404–1.127	0.1640			
HBV DNA (log_10_ IU/mL) (>4, 24 vs. ≤4, 12)	2.029	0.733–5.618	0.1731			
Albumin (g/dL) (>3.8, 18 vs. ≤3.8, 22)	0.361	0.152–0.858	0.0211 *	0.309	0.113–0.845	0.0211 *^,c^
				0.323	0.113–0.921	0.0345 *^,d^
				0.322	0.117–0.887	0.0284 *^,e^
				0.321	0.113–0.914	0.0333 *^,f^
				0.322	0.117–0.887	0.0284 *^,g^
				0.345	0.119–1.000	0.0501 ^h^
				0.293	0.103–0.835	0.0216 *^,i^
				0.345	0.118–1.011	0.0524 ^j^
AST (U/L) (>34, 32 vs. ≤34, 8)	1.619	0.546–4.802	0.3851			
ALT (U/L) (>40, 26 vs. ≤40, 14)	2.910	1.083–7.824	0.0342 *	2.163	0.746–6.269	0.1555 ^c^
				2.060	0.703–6.035	0.1874 ^d^
				2.084	0.721–6.018	0.1749 ^e^
				1.961	0.680–5.654	0.2127 ^f^
				2.084	0.721–6.018	0.1749 ^g^
				1.858	0.645–5.256	0.2512 ^h^
				1.897	0.658–5.468	0.2357 ^i^
				1.929	0.625–5.955	0.2535 ^j^
AFP (ng/mL) (>400, 15 vs. ≤400, 25)	2.805	1.261–6.240	0.0114 *	2.750	1.057–7.153	0.0381 *^,c^
				2.596	0.964–6.989	0.0590 ^d^
				2.570	0.970–6.813	0.0577 ^e^
				2.664	0.987–7.185	0.0530 ^f^
				2.570	0.970–6.813	0.0577 ^g^
				2.411	0.862–6.750	0.0937 ^h^
				2.857	1.042–7.832	0.0414 *^,i^
				2.306	0.809–6.572	0.1181 ^j^
Tumor size (cm) (>5, 14 vs. ≤5, 26)	0.900	0.387–2.093	0.8070			
Tumor encapsulation (yes, 30 vs. no, 9)	0.775	0.305–1.969	0.5924			
Satellite nodule (yes, 7 vs. no, 33)	1.539	0.566–4.189	0.3984			
Lymph node involvement (yes, 6 vs. no, 34)	0.550	0.129–2.348	0.4193			
Portal vein thrombosis (yes, 1 vs. no, 39)	3.497	0.908–22.503	0.0928			
Vascular invasion (yes, 18 vs. no, 22)	1.807	0.807–4.044	0.1504			
Distant metastasis (yes, 4 vs. no, 36)	1.586	0.467–5.392	0.4597			
Steatosis grade (2/3, 7 vs. 0/1, 33)	3.422	0.427–27.393	0.2464			
Metavir inflammation score (2/3, 1 vs. 0/1, 34)	0.921	0.122–6.943	0.9360			
Ishak fibrosis score (4/5/6, 15 vs. 0/1/2/3, 24)	1.879	0.824–4.287	0.1337			
Child-Pugh cirrhosis score (B/C, 10 vs. A, 30)	2.715	1.151–6.406	0.0226 *	1.442	0.549–3.788	0.4573 ^c^
				1.315	0.499–3.471	0.5796 ^d^
				1.383	0.522–3.667	0.5146 ^e^
				1.360	0.519–3.561	0.5311 ^f^
				1.383	0.522–3.667	0.5146 ^g^
				1.280	0.482–3.397	0.6199 ^h^
				1.213	0.462–3.184	0.6946 ^i^
				1.201	0.446–3.234	0.7170 ^j^
CLIP score (4/5/6, 2 vs. 0/1/2/3, 38)	1.088	0.146–8.124	0.9342			
BCLC stage (C/D, 8 vs. A/B, 32)	2.243	0.870–5.783	0.0944			
AJCC TNM stage (IIIA/IIIB/IIIC/IVA/IVB, 9 vs. I/II, 31)	2.486	1.003–6.162	0.0492 *	1.607	0.521–4.958	0.4096 ^c^
				1.613	0.544–4.784	0.3888 ^d^
				1.772	0.547–5.732	0.3399 ^e^
				1.594	0.534–4.757	0.4037 ^f^
				1.772	0.547–5.732	0.3399 ^g^
				1.807	0.574–5.691	0.3123 ^h^
				1.677	0.533–5.276	0.3769 ^i^
				1.953	0.580–6.576	0.2800 ^j^

^a^ Only patients with available data were analyzed. ^b^ *p* value was determined by the Cox proportional-hazards regression model. ^c,d,e,f,g,h,i,j^ Multivariate analysis was performed between the clinicopathological characteristics labeled with the same symbols. *, *p* value < 0.05; **, *p* value < 0.01. Abbreviations: HBV, hepatitis B virus; HCC, hepatocellular carcinoma; HR, hazard ratio; CI, confidence interval; PD-L1, programmed death ligand 1; Tregs, regulatory T cells; vs., versus; HBeAg, hepatitis B e antigen; AST, aspartate aminotransferase; ALT, alanine aminotransferase; AFP, alpha-fetoprotein; CLIP, Cancer of the Liver Italian Program; BCLC, Barcelona Clinic Liver Cancer; AJCC, American Joint Committee on Cancer; TNM, tumor-node-metastasis.

## Data Availability

All relevant data are within the paper and its supplementary materials.

## Supplementary Materials

The following supporting information can be downloaded at: https://www.mdpi.com/article/10.3390/v14061346/s1, Table S1: Pre-S2 gene deletion regions of the 21 HBV pre-S2 mutant-positive HCC patients; Figure S1: Representative images of PD-L1-expressing cells and Tregs in fluorescent IHC-stained HCC Tissues.
