# Supplementary material for: Association of Increased Programmed Death Ligand 1 Expression and Regulatory T Cells Infiltration with Higher Hepatocellular Carcinoma Recurrence in Patients with Hepatitis B Virus Pre-S2 Mutant after Curative Surgical Resection
Source: Viruses. 2022 Jun 20;14(6):1346. doi: 10.3390/v14061346 (PMC9229682; doi:10.3390/v14061346)
Supplement: Supplementary file 1 [file viruses-14-01346-s001.zip › Table S1.pdf]

Table S1. Pre-S2 gene deletion regions of the 21 HBV pre-S2 mutant-positive HCC patients

Patient No.	Pre-S2 Gene Deletion Region (%)[a]	Deletion Size
1	nucleotides 24-50 (33.801)	27 nucleotides
2	nucleotides 1-54 (11.589)	54 nucleotides
3	nucleotides 1-54 (27.093)	54 nucleotides
4	nucleotides 1-54 (63.181)	54 nucleotides
5	nucleotides 1-51 (11.029)	51 nucleotides
6	nucleotides 1-54 (53.898)	54 nucleotides
7	nucleotides 1-54 (12.523)	54 nucleotides
8	nucleotides 1-57 (10.490)	57 nucleotides
9	nucleotides 1-54 (23.226)	54 nucleotides
10	nucleotides 1-54 (10.226)	54 nucleotides
11	nucleotides 1-54 (26.027)	54 nucleotides
12	nucleotides 1-15 (11.710)	15 nucleotides
13	nucleotides 1-54 (51.071)	54 nucleotides
14	nucleotides 15-56 (33.789)	42 nucleotides
15	nucleotides 1-57 (22.473)	57 nucleotides
16	nucleotides 1-54 (15.565)	54 nucleotides
17	nucleotides 1-54 (39.358)	54 nucleotides
18	nucleotides 1-54 (38.244)	54 nucleotides
19	nucleotides 1-54 (13.399)	54 nucleotides
20	nucleotides 1-54 (23.550)	54 nucleotides
21	nucleotides 1-57 (20.926)	57 nucleotides

[a]The pre-S2 gene deletion region with the highest frequency in each patient was shown.
