# Supplementary figures and images for: Association of Increased Programmed Death Ligand 1 Expression and Regulatory T Cells Infiltration with Higher Hepatocellular Carcinoma Recurrence in Patients with Hepatitis B Virus Pre-S2 Mutant after Curative Surgical Resection
Source: Viruses. 2022 Jun 20;14(6):1346. doi: 10.3390/v14061346 (PMC9229682; doi:10.3390/v14061346)

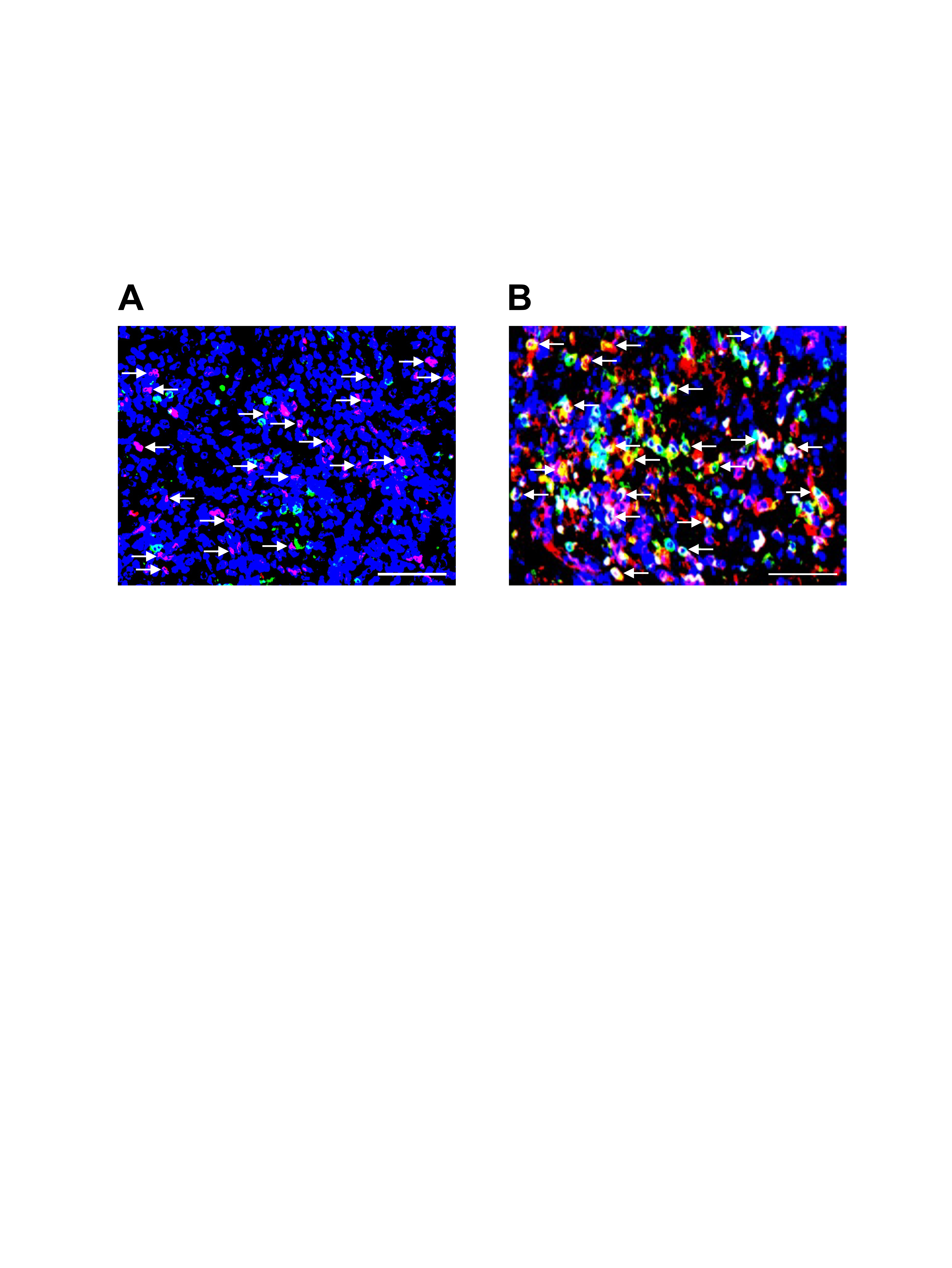

Supplement: Supplementary file 1 [file viruses-14-01346-s001.zip › Figure S1.tif]
